# Pulmonary Function and Radiographic Outcomes in Systemic Sclerosis-Associated Interstitial Lung Disease: A Systematic Review of Rituximab, Interleukin-6 Inhibitors, and Janus Kinase Inhibitors

**DOI:** 10.7759/cureus.112918

**Published:** 2026-07-18

**Authors:** Rohan M Patel, Nidhi Chary, Marc M Kesselman

**Affiliations:** 1 Osteopathic Medicine, Dr. Kiran C. Patel College of Osteopathic Medicine, Nova Southeastern University, Fort Lauderdale, USA; 2 Medicine, Dr. Kiran C. Patel College of Osteopathic Medicine, Nova Southeastern University, Fort Lauderdale, USA; 3 Rheumatology, Dr. Kiran C. Patel College of Osteopathic Medicine, Nova Southeastern University, Davie, USA

**Keywords:** interleukin-6 inhibitors, janus kinase inhibitor, pulmonary fibrosis, rituximab (rtx), systemic sclerosis interstitial lung disease (ssc-ild)

## Abstract

Systemic sclerosis-associated interstitial lung disease (SSc-ILD) is a major cause of morbidity and mortality in systemic sclerosis patients. Although management typically includes conventional immunosuppression, increasing attention has been given to targeted immunomodulatory therapies including rituximab (RTX), IL-6 inhibitors (IL-6i), and JAK inhibitors (JAKi). This systematic review aimed to synthesize the available evidence on target immunomodulation as a management strategy for SSc-ILD, with a focus on post-exposure pulmonary function testing and radiographic progression. A systematic literature review was conducted utilizing EMBASE, Ovid MEDLINE, PubMed, and Web of Science in accordance with Preferred Reporting Items for Systematic Reviews and Meta-Analyses (PRISMA) 2020 guidelines. The targeted population included adult patients with either SSc-ILD or extractable pulmonary outcomes. Outcomes of interest included forced vital capacity (FVC), diffusing capacity for carbon monoxide (DLCO), total lung capacity (TLC), and high-resolution computed tomography (HRCT) progressive outcomes. Twenty-three studies met the inclusion criteria. FVC was the most consistently reported pulmonary outcome, while DLCO, TLC, and serial HRCT outcomes were less uniformly assessed. Due to substantial heterogeneity within the selected studies, a meta-analysis was not performed; findings were synthesized narratively. Targeted immunomodulatory therapy was more commonly associated with stabilization of pulmonary function rather than with substantial improvement. Tocilizumab demonstrated the strongest evidence for relative FVC preservation in placebo-controlled studies while RTX showed recurrent stabilization signals across comparative and observational studies. Evidence for JAKi remained premature, with limited pulmonary function data and inconsistent outcome reporting. Substantial heterogeneity in study design, patient populations, concomitant immunosuppression, comparators, and outcome reporting limited direct comparison across the therapies evaluated. Future research should account for inconsistencies amongst studies, namely population sizes, concurrent therapies, study types, and comparison groups. Future prospective studies using standardized criteria, outcome definitions, and comparator groups are needed to improve the causal interpretation.

## Introduction and background

Systemic sclerosis is an autoimmune connective tissue disease accompanied by immune dysregulation, vasculopathy, and progressive cutaneous and visceral fibrosis [1]. Systemic sclerosis-associated interstitial lung disease (SSc-ILD) remains a critically consequential complication when discussing the visceral involvement of the disease, contributing to the functional limitation, premature mortality, and significant morbidity these patients experience [2]. Pulmonary involvement may occur early in the disease course and can range from minute radiographic abnormalities to progressive fibrosing disease marked by a decrease in forced vital capacity (FVC), impaired diffusing capacity for carbon monoxide (DLCO), and radiographic progression on high-resolution computed tomography (HRCT). Clinically, this manifests as patients with recurrent respiratory distress [3]. Due to the progressive nature of the disease, potentially causing irreversible pulmonary decline once advanced fibrosis develops, prompt recognition and disease-modifying therapy are pivotal to SSc-ILD prognostic outcomes [4]. 

Thus far, treatment of SSc-ILD has relied heavily on conventional immunosuppressive therapies, notably cyclophosphamide (CY) and mycophenolate mofetil (MMF). The Scleroderma Lung Study I demonstrated that CY could improve or stabilize pulmonary function when compared to placebo groups, although the benefits were ultimately limited; later, the Scleroderma Lung Study II supported MMF as a better-tolerated alternative with comparable efficacy [5]. More recently, rheumatologists have been entertained by the novel development of antifibrotic therapy. The Safety and Efficacy of Nintedanib in Systemic Sclerosis (SENSCIS) trial has shown that nintedanib, an antifibrotic tyrosine kinase inhibitor, reduced the rate of FVC decline in SSc-ILD patients, demonstrating the potential role of fibrotic pathways in SSc-ILD progression and supporting the notion that there may be effective therapies to slow disease progression beyond conventional immunosuppressive therapies alone [6]. Despite such advances, therapeutic uncertainty remains. Furthermore, SSc-ILD treatment selection is surely complicated by the heterogeneity in disease phenotype, fibrosis burden, serologic profile, and medication tolerability [7]. There is growing interest in utilizing target immunomodulatory therapies to address specific immune pathways implicated in the pathogenesis of SSc-ILD, including B-cell activation, interleukin-6 (IL-6) signaling, and Janus kinase (JAK) signaling [8]. Because pulmonary fibrosis appears to arise from the interaction of inflammatory, vascular, and fibroproliferative mechanisms, targeting these pathways may be particularly relevant for slowing disease progression [9]. 

Rituximab (RTX), a monoclonal antibody targeting CD20-positive B cells, has been evaluated in numerous SSc-ILD and connective tissue disease-associated ILD (CT-ILD) studies. The Safety and Efficacy of Rituximab for Systemic Sclerosis (DESIRES) trial has suggested potential benefits in pulmonary and dermatologic outcomes among patients with SSc-ILD [10]. The Rituximab versus Cyclophosphamide for the Treatment of Connective Tissue Disease-Associated Interstitial Lung Disease (RECITAL) trial compared RTX to CY in CT-ILD and also reported improvements in pulmonary function tests (PFTs) and drug tolerability [11]. IL-6 inhibitors (IL-6i) have also emerged as a potential therapeutic for SSc-ILD. The faSScinate and focuSSced trials have demonstrated preservation of FVC in patients with ILD secondary to administration of tocilizumab (TCZ) [12,13]. However, the extent to which IL-6 inhibition reduces fibrotic disease progression, compared to conventional immunomodulatory therapy, remains unclear. JAK inhibitors (JAKi) are a newer and less established class amongst the therapeutics involved in SSc-ILD management. Considering the role that cytokine signaling pathways play in immune activation and fibrotic remodeling, JAKi theoretically should have a plausible role in SSc-ILD management [14]. The role of JAKi in SSc-ILD remains investigational and surely requires further evaluation. 

Although individual studies have begun to examine RTX, IL-6i, and JAKi in the management of SSc-ILD, the evidence remains heterogeneous. That is, existing studies differ significantly in study design, sample size, diagnostic criteria, comparator groups, and concomitant immunosuppression. Pulmonary endpoints are also variably reported across studies, with some only reporting FVC, others DLCO, HRCT progression, or composite clinical outcomes. Inconsistencies such as these limit direct comparative ability and make it difficult to determine how these targeted therapies should be incorporated into SSc-ILD treatment algorithms. The purpose of this systematic review is to synthesize the available evidence on novel targeted immunomodulatory therapies in SSc-ILD. By evaluating B-cell targeted therapy, IL-6i, and JAKi in adults with SSc-ILD, this review aims to clarify the role of targeted immunomodulation in SSc-ILD, identify areas of uncertainty, and highlight gaps that can guide clinical trials and comparative research in the future. 

## Review

Methods 

Study Design & Protocol Registration

This review was conducted and reported in accordance with the Preferred Reporting Items for Systematic Reviews and Meta-Analyses (PRISMA) 2020 guidelines [15]. The protocol was preemptively registered in the International Prospective Register of Systematic Reviews (PROSPERO) under registration number CRD420261357125 [16]. No amendments to the protocol were made after registration. 

Eligibility Criteria 

Studies were eligible for inclusion if they evaluated adult patients (>18 years old) with SSc-ILD and reported outcomes following treatment with immunomodulatory therapies. Studies that enrolled broader systemic sclerosis populations were included only when SSc-ILD subgroup data, baseline ILD status, or pulmonary outcomes relevant to ILD progression were extractable. Therapies of interest included B-cell targeted therapy, IL-6i, and JAKi. Peer-reviewed human studies published in 2015 and onward were included. Eligible studies included randomized controlled trials, prospective and retrospective cohort studies, case-control studies, and other observational studies that reported relevant clinical, pulmonary function, or radiographic outcomes. Case reports, case series, reviews, meta-analyses, editorials, letters, non-human studies, and conference abstracts without full-text outcome data available were excluded. Points of comparison amongst studies included baseline pulmonary function testing, radiographic findings, placebo therapy, or conventional treatment with CY, MMF, or corticosteroids. The primary outcomes of interest included changes in pulmonary function testing, radiographic changes seen on high-resolution computed tomography (HRCT), mortality, or related measures of ILD progression and treatment response. Since eligible studies included both observational and interventional studies, a PECO framework was used rather than the traditional PICO framework, conceptualizing RTX, IL-6i, and JAKi as the therapeutic exposures, while allowing the comparator group to include baseline values, placebo, standard of care, or active treatment (Table 1). 

**Table 1:** PECO framework for systematic reviews SSc-ILD: systemic sclerosis-associated interstitial lung disease; HRCT: high-resolution computed tomography; ILD: interstitial lung disease; IL-6: interleukin-6; JAK: Janus kinase; FVC: forced vital capacity; DLCO: diffusion capacity of the lungs for carbon monoxide; TLC: total lung capacity

Framework	Parameters
Population	Adults aged ≥18 years with SSc-ILD, diagnosed clinically and/or by HRCT evidence of ILD, pulmonary function abnormalities consistent with ILD, or multidisciplinary diagnosis
Exposure	Targeted immunomodulatory therapy for SSc-ILD, including B-cell-targeted therapy such as rituximab, IL-6 inhibitors such as tocilizumab or sarilumab, and JAK inhibitors such as tofacitinib, baricitinib, or upadacitinib
Comparator	Placebo, conventional therapy such as cyclophosphamide, mycophenolate mofetil, or corticosteroids, or within-cohort baseline pulmonary function/radiographic measurements for pre- and post-treatment comparison
Outcome	Pulmonary function outcomes, including FVC, DLCO, and TLC; radiographic outcomes, including HRCT findings or ILD progression; clinical outcomes, including treatment discontinuation

Search Strategy

A comprehensive literature search was performed across EMBASE, Ovid MEDLINE, PubMed, and Web of Science. The search strategy was developed to recognize peer-reviewed published studies evaluating targeted immunomodulatory therapies in SSc-ILD. Free-text and controlled search terms related to systemic sclerosis, scleroderma, ILD, pulmonary fibrosis, immunomodulatory therapy, RTX, B-cell therapy, IL-6 therapy, JAKi therapy, and related agents were used (Appendices A-D). Search results from all databases were exported and compiled. The final searches were conducted on April 2nd, 2026. 

Study Selection and Screening

The comprehensive literature search yielded 2,435 records, including 1,487 from EMBASE, 273 from Ovid MEDLINE, 240 from PubMed, and 435 from Web of Science, all of which were uploaded into the Rayyan systematic review collaboration software (Rayyan Systems Inc., Cambridge, MA, US). After the removal of 1,343 duplicate records, 1,092 records remained. The screening process was conducted in two distinct tiers. During tier one screening, titles and abstracts were independently screened in a blinded manner by two reviewers (RP, NC) using the preconceived eligibility criteria, screening articles for advancement to full-text review. Conflicts at this stage were resolved through discussion. Of the 1,092 records screened, 962 were excluded due to ineligibility based on publication date, study design, publication type, population intervention, or relevance. A total of 130 reports were sought for retrieval, of which 23 were unretrievable. The remaining 107 reports were further assessed in tier two. During tier two screening, full-text articles were independently reviewed in a blinded manner by both reviewers (RP, NC) using the same eligibility criteria. Diagreements regarding full-text eligibility were resolved through discussion and consensus. No third reviewer was required for decision adjudication. 

**Figure 1:**
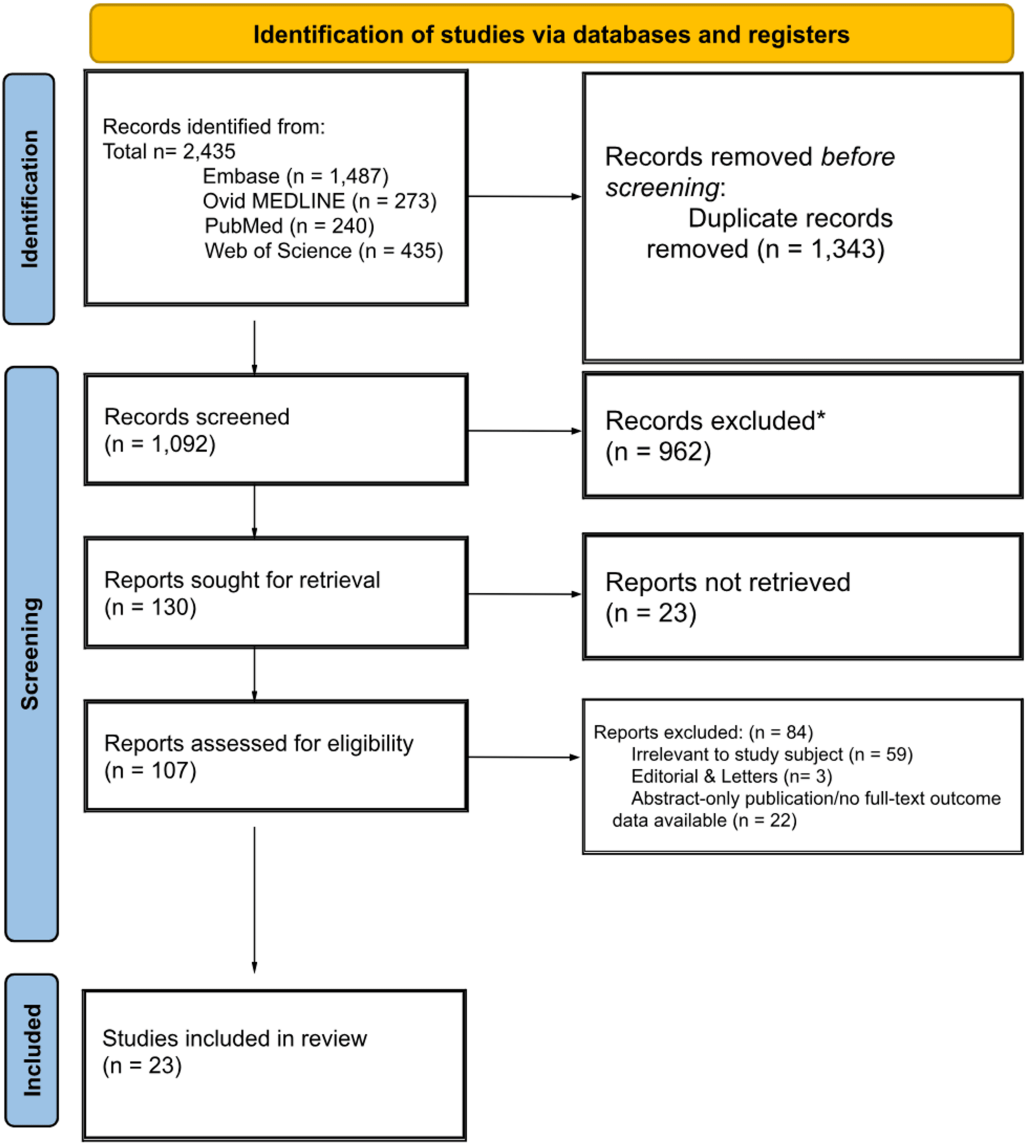
PRISMA flow diagram *Records excluded during title/abstract screening were ineligible based on publication date, publication type, study design, population, intervention, or relevance to the review question.

Twenty-three studies met the inclusion criteria and were included in the systematic review. No study authors were contacted for missing data, eligibility clarification, or outcome information not available within the full text. A PRISMA flow diagram was used to summarize the study identification, screening, eligibility assessment, and ultimate inclusion process (Figure 1). 

Data Extraction

Data was extracted using a standardized data chart in Google Sheets (Google LLC; Mountain View, CA, USA). Study characteristics extracted included author, year of publication, country of origin, study design, sample size, intervention and comparator group details, baseline, and post-treatment pulmonary function (TLC, FVC, DLCO) and radiographic findings. Pertinent safety outcomes were also noted. Numerical values were extracted exactly as reported; that is, mean values were extracted with standard deviations, and median values were extracted with interquartile ranges when provided. Dispersion metrics were not combined or converted. P-values were extracted only when clearly associated with the outcome being reported. If a study stated that there was no significant difference but did not provide an exact p-value, this was recorded as “NR.” In a separate table, also using Google Sheets, study-specific systemic sclerosis and ILD definitions were listed as reported in the original papers, due to heterogeneity and varied classification criteria (Table 2).

**Table 2:** Study-specific SSc and ILD definitions ACR: American College of Rheumatology; EULAR: European League Against Rheumatism; ARA: American Rheumatism Association; HRCT: high-resolution computed tomography; ILD: interstitial lung disease; SSc: systemic sclerosis; PFT: pulmonary function test; FVC: forced vital capacity; DLCO: diffusing capacity of the lungs for carbon monoxide; CTD: connective-tissue disease; NSIP: nonspecific interstitial pneumonia; NR: not reported.

Study	Study-specific SSc diagnostic criteria (if provided)	Study-specific ILD diagnostic criteria (if provided)
Hiura et al., 2025 [17]	2013 ACR/EULAR classification criteria	ILD diagnosed by a cardiothoracic radiologist based on HRCT findings. The NSIP pattern was determined based on the 2013 ATS/ERS international classification. SSc-ILD was further classified as extensive or limited disease based on the Goh staging system
Barde et al., 2025 [18]	2013 ACR/EULAR classification criteria	PFTs values, including predicted FVC and DLCO, plus clinical assessment
Goldman et al., 2025 [19]	2013 ACR/EULAR diagnostic criteria for systemic sclerosis	HRCT performed within 12 months before biologic initiation, scored using the Goh extent-of-disease staging system by two independent assessors; extensive disease was defined as ≥20% involvement.
Yan et al., 2025 [20]	Either the 2013 ACR/EULAR classification criteria or the 1980 ACR classification	Labelled in the EUSTAR database as having HRCT or X-ray-confirmed ILD. HRCT features recorded included ground glass, honeycombing, and reticular change.
Moazedi-Fuerst et al., 2024 [21]	LeRoy criteria and ACR/EULAR classification criteria (unspecified year). All patients underwent capillaroscopy showing an SSc pattern, such as megacapillaries.	Inflammatory/fibrotic lung involvement confirmed by HRCT, including ground-glass opacities, reticular pattern, and NSIP pattern. Lung disease progression was defined as DLCO decline >15% predicted and/or HRCT progression >10% parenchymal involvement. HRCT involvement was categorized as mild <10%, moderate 10–20%, or severe >20% lung parenchymal change.
De Luca et al., 2025 [22]	2013 ACR/EULAR classification criteria	ILD diagnosed on HRCT
Ghuman et al., 2024 [23]	focuSSced cohort: 2013 ACR/EULAR classification criteria, disease duration ≤60 months from first non-Raynaud manifestation, and mRSS 10-35 at screening. SMART cohort: Patients from the Royal Free Scleroderma Cohort with systemic sclerosis	focuSSced cohort: ILD identified on baseline HRCT visual read by an experienced thoracic radiologist SMART cohort: Patients had HRCT-confirmed ILD based on visual HRCT read
Junfei et al., 2023 [24]	2013 ACR/EULAR classification criteria	Chest HRCT performance, using basic CT terminology from the Fleischner Society glossary. HRCT abnormalities were classified as early exudative lesions, early fibrosis/middle-stage lesions, or later extensive fibrosis. Pulmonary impairment was scored using the Warrick semi-quantitative HRCT scoring method
Kuzumi et al., 2023 [10]	2013 ACR/EULAR classification criteria	ILD/pulmonary fibrosis was identified on HRCT. The article cites the Goh et al. SSc-ILD staging system in relation to pulmonary fibrosis/prognosis, but does not provide a study-specific HRCT scoring definition
Maher et al., 2023 [11]	2013 ACR/EULAR classification criteria	Severe or progressive ILD related to CTD and an HRCT within 12 months before randomization, documenting evidence of ILD. Severity/progression determination was left to investigators
Yalcinkaya et al., 2023 [25]	2013 ACR/EULAR classification criteria	Pulmonary fibrosis/progression was detected using HRCT of the chest and pulmonary function tests together
Kuster et al., 2022 [26]	2013 ACR/EULAR classification criteria	Evidence of ILD on HRCT or X-ray, as judged by the local investigator. Lung progression/regression was defined using FVC and DLCO thresholds- decline/increase in either FVC ≥10% or FVC ≥5% with DLCO ≥15%
Isomura et al., 2022 [27]	ACR/EULAR classification criteria (unspecified year)	ILD confirmed by HRCT, no further specifications in paper
Khanna et al., 2022 [28]	2013 ACR/EULAR classification criteria	ILD evidence was not required
Yilmaz et al., 2021 [29]	1980 ARA Criteria or 2013 ACR/EULAR classification criteria	NR
Roofeh et al., 2021 [12]	2013 ACR/EULAR classification criteria	NR
Robles-Perez et al., 2020 [30]	Multidisciplinary diagnosis following standard guidelines	NR
Sircar et al, 2018 [31]	2013 ACR/EULAR classification criteria	HRCT and PFT (FVC at least 45% but <80% and reproducible within 10% at baseline appointment)
Daoussis et al., 2017 [32]	2013 ACR/EULAR classification criteria	SSc-ILD was documented with PFTs and HRCT, FVC and/or DLCO <80% of predicted values, and HRCTs demonstrated ground glass, honeycombing, or reticular lesions by an experienced radiologist.
Lepri et al., 2016 [33]	2013 ACR/EULAR classification criteria	Ground-glass or reticular pattern or honey combing on HRCT
Jordan et al., 2015 [34]	2013 ACR/EULAR classification criteria	FVC <70% predicted and evidence for lung fibrosis on HRCT at baseline
Khanna et al., 2016 [35]	1980 ACR criteria	NR
Kuwana et al., 2024 [36]	2013 ACR/EULAR classification criteria	HRCT read by thoracic radiologist

Most included studies used the 2013 American College of Rheumatology/European League Against Rheumatism (ACR/EULAR) classification criteria for systemic sclerosis, while others used older ACR criteria, LeRoy criteria, or broader internationally accepted diagnostic criteria. Diagnostic criteria was extracted as reported by each study. 

Risk of Bias Assessment

Two reviewers independently completed the risk of bias assessment. Randomized controlled trials were assessed using the Cochrane Risk-of-Bias Tool for RCTs Version 2 (RoB 2), and observational studies were assessed using the Newcastle-Ottawa Scale (NOS) [37,38]. Each study received an overall judgment of low risk, some concerns (RoB 2)/moderate risk (NOS), or high risk of bias. Domain-level findings were combined, according to their respective frameworks, to enhance interpretability and organization. RoB findings were summarized descriptively (Table 3). 

**Table 3:** Risk of Bias assessment For the Newcastle-Ottawa Scale (NOS), higher scores represent a lower risk of bias. Overall, NOS judgments were categorized according to the predefined NOS score thresholds. RoB 2 studies were judged using domain-level assessments according to the Cochrane RoB 2 framework. Because NOS and RoB 2 assess different study designs, domain headings were harmonized for display but interpreted according to the respective tools.

Study	Scale used	Randomization process/selection	Comparability/deviations from intended interventions	Outcome measurement	Missing outcome data	Selection of the reported result	Overall Risk of Bias
Hiura et al., 2025 [17]	NOS	3	2	3	N/A	N/A	Low risk
Barde et al., 2025 [18]	NOS	3	1	3	N/A	N/A	Low risk
Goldman et al., 2025 [19]	NOS	3	1	2	N/A	N/A	Moderate risk
Yan et al., 2025 [20]	NOS	4	2	3	N/A	N/A	Low risk
Moazedi-Fuerst et al., 2024 [21]	NOS	3	0	2	N/A	N/A	Moderate risk
De Luca et al., 2025 [22]	NOS	3	0	2	N/A	N/A	Moderate risk
Ghuman et al., 2024 [23]	RoB 2	Low risk	Low risk	Low risk	Low risk	Some concerns	Some concerns
Junfei et al., 2023 [24]	NOS	3	1	2	N/A	N/A	Moderate risk
Kuzumi et al., 2023 [10]	NOS	3	0	2	N/A	N/A	Moderate risk
Maher et al., 2023 [11]	RoB 2	Low risk	Low risk	Low risk	Some concerns	Low risk	Some concerns
Yalcinkaya et al., 2023 [25]	NOS	3	0	2	N/A	N/A	Moderate risk
Kuster et al., 2022 [26]	NOS	4	2	3	N/A	N/A	Low risk
Isomura et al., 2022 [27]	NOS	3	0	2	N/A	N/A	Moderate risk
Khanna et al., 2022 [28]	RoB 2	Low risk	Some concerns	Low risk	Low risk	Low risk	Some concerns
Yilmaz et al., 2021 [29]	NOS	3	1	2	N/A	N/A	Moderate risk
Roofeh et al., 2021 [12]	RoB 2	Low risk	Low risk	Low risk	Low risk	Low risk	Low risk
Robles-Perez et al., 2020 [30]	NOS	2	0	2	N/A	N/A	Moderate risk
Sircar et al., 2018 [31]	RoB 2	Low risk	Some concerns	Some concerns	Low risk	Low risk	Some concerns
Daoussis et al., 2017 [32]	NOS	3	1	1	N/A	N/A	Moderate risk
Lepri et al., 2016 [33]	NOS	3	0	1	N/A	N/A	Moderate risk
Jordan et al., 2015 [34]	NOS	3	1	2	N/A	N/A	Moderate risk
Khanna et al., 2016 [35]	RoB 2	Low risk	Low risk	Low risk	Low risk	Low risk	Low risk
Kuwana et al., 2024 [36]	RoB 2	Low risk	Low risk	Low risk	Low risk	Low risk	Low risk

Data Synthesis

This study was designed as a systematic review with narrative synthesis; that is, a quantitative meta-analysis was not planned due to anticipated heterogeneity across studies, including differences in study design, patient populations, SSc-ILD study-specific definitions, immunomodulatory treatments administered, follow-up intervals, pulmonary function outcomes, radiographic endpoints, and outcome reporting. Findings were synthesized descriptively and organized by clinical outcomes, rather than being pooled statistically. Pulmonary outcomes were summarized according to the magnitude of effect on pulmonary function tests, and radiographic findings were explained. When both unadjusted and adjusted analyses were reported, adjusted analyses were prioritized for better interpretation, and unadjusted values were reported when adjusted values were unavailable. Findings were interpreted in the context of study design, risk of bias, sample size, and consistency across studies. 

Results

A total of 23 studies were included in this systematic review after tier one and tier two screening. Included studies evaluated SSc-ILD immunomodulatory therapies, with the most common interventions being RTX and TCZ, with fewer studies assessing JAKi. Study designs were grouped according to primary analytic design, classified as randomized controlled trials (n=6), retrospective cohort/observational studies (n=14), and prospective cohort/observational studies (n=3). Across the studies, clinical outcomes were assessed using PFTs, most commonly FVC and DLCO. Fewer studies reported TLC or serial HRCT progressive outcomes. Due to the heterogeneity in study design, outcome reporting, therapeutic exposure, and reporting format, results were synthesized narratively rather than pooled quantitatively. The individual study characteristics and outcomes are summarized in Table 4. 

**Table 4:** Results from included studies SSc-ILD: systemic sclerosis-associated interstitial lung disease; HRCT: high-resolution computed tomography; ILD: interstitial lung disease; IL-6: interleukin-6; JAK: Janus kinase; FVC: forced vital capacity; DLCO: diffusion capacity of the lungs for carbon monoxide; TLC: total lung capacity; RTX: rituximab; TCZ: tocilizumab; CY: cyclophosphamide; MMF: mycophenolate mofetil; DMARDs: disease-modifying antirheumatic drugs; ATA: anti-topoisomerase antibody; IV: intravenous; SC: subcutaneous; NR: not reported; RECITAL: Rituximab versus Cyclophosphamide for the Treatment of Connective Tissue Disease-Associated Interstitial Lung Disease; DESIRES: Safety and Efficacy of Rituximab for Systemic Sclerosis.

Study	Sample size	Intervention and route (if available)	Comparators (if available)	Main pulmonary findings	Statistical significance	Overall interpretation
Hiura et al., 2025 [17]	50 total: RTX n=20, CY n=30	IV RTX 365 mg/m² per dose, 4 doses 1 week apart	IV CY 500 mg/m², 6 doses every 4 weeks	FVC improved numerically in both groups at six and 12 months. HRCT/PPF progression occurred in 3/20 RTX patients and 5/30 CY patients	FVC change didn't significantly differ between RTX and CY at 6 months (p=0.59) or 12 months (p=0.74). HRCT/PPF progression HR 0.91, 95% CI 0.24–3.37, p=0.52	RTX and CY showed comparable pulmonary stabilization, with no significant difference in FVC change or HRCT/PPF progression
Barde et al., 2025 [18]	127 total; MMF/RTX n=47, RTX n=30, MMF n=50	MMF/RTX combination therapy	RTX monotherapy and MMF monotherapy	The MMF/RTX Combination group showed FVC improvement from 63.1% (54.3-73.8) predicted to 67.0% (57.5-80.5) predicted at 12 months. DLCO improved numerically from 38.0% predicted (31.0-52.0) to 43.5% (31.0-57.0) predicted. HRCT progression could not be evaluated due to missing HRCT data	Within-group MMF/RTX FVC improvement was significant (p=0.002) but not RTX monotherapy (p=0.8) or MMF monotherapy (p=0.5). DLCO changes were not significant (MMF/RTX p=0.2, RTX p=0.8, MMF p=0.6)	The MMF/RTX combination was associated with significant FVC improvement within the group, whereas RTX or MMF monotherapy did not show significant FVC change. HRCT conclusions were limited by missing imaging data. Because between-group data was restricted, causal association cannot be made
Goldman et al., 2025 [19]	127 total; RTX n=87, TCZ n=32, combined RTX/TCZ n=8	RTX and TCZ biologic therapy	No untreated control- comparison of cohorts and pre/post biologic	Both RTX and TCZ were associated with stabilization or improvement in FVC and DLCO trajectories after biologic initiation. TCZ showed greater numerical FVC response among anti-topoisomerase I antibody-positive (ATA) patients	ATA positivity was associated with greater post-treatment FVC response to TCZ (p=0.05, 95% CI 0.02-10.6 initially); after adjusting, p=0.03, 95% CI 0.45-11.09. ATA-positive patients had numerically greater but not statistically significant %FVC response to RTX	TCZ benefit may be greater in ATA-positive patients, but findings are observational
Yan et al., 2025 [20]	955 total; TCZ n=96, RTX n=172, MMF n=511, CY n=218	TCZ, RTX, MMF, and CY (dosing NR)	IPTW-adjusted comparisons among treatment groups with background immunosuppressant, antifibrotic, and corticosteroid use accounted for	At approximately one year, FVC % predicted change did not significantly differ among TCZ, RTX, MMF, and CY. DLCO % predicted change also did not significantly differ among groups	FVC % predicted change p=0.101; DLCO % predicted change p=0.227. Paired average treatment effect comparisons were nonsignificant.	In IPTW-adjusted registry analysis, TCZ, RTX, MMF, and CY had comparable pulmonary outcomes at approximately 1 year and no therapeutic superiority was established
Moazedi-Fuerst et al., 2024 [21]	40 total	Long-term low-dose RTX regimen, commonly 500 mg at day zero and 14 every 3 months	No untreated control; RTX monotherapy and RTX + background immunosuppression were compared descriptively	FVC remained stable during the first year of long-term low dose RTX therapy, with no reported FVC decline during treatment. DLCO slightly but nonsignificantly increased during the first year before plateauing	No significant FVC or DLCO difference over time (exact p-values NR) in RTX monotherapy or RTX/MMF groups	Long-term low-dose RTX was associated with nonsignificant stabilization of FVC and nonsignificant DLCO improvement, but lack of comparator limits interpretation
De Luca et al., 2025 [22]	152 total; 115 with SSc-ILD	RTX 1000 mg repeated after 15 days; repeated doses were common	No untreated controls. Concomitant DMARDs/steroids/nintedanib permitted	In the ILD subgroup, FVC remained stable from baseline (81.6±17.8% predicted) to six months (84.7±19.2% predicted) and final follow-up (82.5±21.6% predicted). DLCO remained generally stable from 60.6±18.7% predicted at baseline to 64.5±19.0% predicted at six months and 57.9±18.5% predicted at final follow-up	FVC and DLCO changes were not statistically significant (exact p-values NR)	RTX was associated with long-term stabilization of pulmonary function in SSc-ILD, though no comparator group, no statistical provision, and background therapy limit the interpretation solely to RTX
Ghuman et al., 2024 [23]	focuSSced subgroup: 136 with SSc-ILD; TCZ n=68, placebo n=68	TCZ 162 mg subcutaneously weekly for 48 weeks	Placebo during the focuSSced trial; SMART cohort used for natural history comparison	In the focuSSced ILD subgroup, estimated mean predicted FVC % change at week 48 was +0.74% with TCZ vs -6.32% with placebo. The week-48 between-group difference in predicted FVC % change favored TCZ by 7.06 percentage points	Weekly FVC % predicted difference p<0.0001; week-48 between-group difference p<0.0001	TCZ was associated with FVC preservation compared with placebo in patients with early SSc-ILD, supporting potential benefit in stabilizing FVC decline over time
Junfei et al., 2023 [24]	44 total; tofacitinib n=9, non-tofacitinib n=35	Tofacitinib added to baseline immunosuppressive therapy	Conventional immunosuppressants or glucocorticoids	Add-on tofacitinib did not significantly improve FVC or TLC compared with non-tofacitinib therapy. FVC % predicted change was -0.23% in the tofacitinib group vs -2.6% in the non-tofacitinib group. DLCO % predicted change was +4.56% vs +0.48%, respectively. TLC % predicted change was +2.12% vs -2.1%, respectively. HRCT/Warrick score improved in the tofacitinib group	FVC % predicted change p=0.376; DLCO % predicted change p=0.145; TLC % predicted change p=0.084. Between-group HRCT/Warrick score change p=0.031	Add-on tofacitinib was associated with significant HRCT improvement but did not significantly improve FVC, DLCO, or TLC compared with controls
Kuzumi et al., 2023 [10]	29 total continuing RTX after DESIRES trial; 24 with pulmonary fibrosis	RTX 375 mg/m² IV weekly for four weeks every six months (DESIRES trial continuation)	No untreated control	After three RTX courses, median FVC % predicted change was +1.85 percentage points (0.13-5.68). DLCO showed no significant change.	FVC change after three RTX courses, p<0.001. Low-IgM subgroup had greater FVC improvement, p=0.03. DLCO exact p-value NR	Continued RTX was associated with sustained FVC improvement after the DESIRES trial, but interpretation is limited by lack of untreated comparator, and the biomarker focus in analysis
Maher et al., 2023 [11]	97 total CTD-ILD: 37 with SSc-ILD; CY n=19, RTX n=18	RTX 1000 mg IV at weeks zero and two	IV CY 600 mg/m² every four weeks for six doses	In the SSc subgroup, the FVC volume change was similar between groups: CY +99±329 mL vs RTX +97±234 mL at week 24, and CY +138±440 mL vs RTX +112±249 mL at week 48. DLCO changes were also similar between groups	FVC between group difference: week 24 p=0.493; week 48 p=0.345. DLCO between-group difference: week 24 p=0.425; week 48 p=0.372	RTX and CY produced similar pulmonary outcomes in the SSc subgroup of the RECITAL trial. Between group differences were insignificant. Because RECITAL enrolled mixed CTD-ILD and the SSc subgroup was small, SSc-ILD-specific conclusions could not be made
Yalcinkaya et al., 2023 [25]	27 total; 19 with pulmonary fibrosis	RTX, two infusions of 1000 mg 15 days apart; repeated at six and 12 month intervals	No untreated control and patients had prior or concomitant standard immunosuppressive therapy use	Among patients with pulmonary fibrosis, FVC increased numerically from 70.7±15.4% predicted to 74.3±19.4% predicted at 12 months. DLCO increased numerically from 51.6±22.9% predicted to 53.8±11.3% predicted at 12 months	FVC change p=0.317; DLCO change p=0.655	RTX was associated with numerical PFT improvement, but changes were not statistically significant. Lack of a comparator group and limits the interpretation
Kuster et al., 2022 [26]	TCZ n=93; controls n=3180 before matching	TCZ, IV or subcutaneous (dosing variably reported)	Matched standard-of-care controls	At 12±3 months, FVC was similar between TCZ and matched controls: TCZ 88.7% predicted (95% CI 83.7-93.7) vs control 87.2% predicted (95% CI 80.8-93.6)	Between-group FVC % predicted difference was 1.5 percentage points (95% CI -6.1-9.1), p=0.70	Real-world TCZ use did not show statistically significant improvement in lung fibrosis outcomes compared with matched controls
Isomura et al., 2022 [27]	15 total; 13/15 had HRCT-confirmed ILD at baseline and 10 ILD patients had follow-up FVC data at 12 months	TCZ therapy followed by continued treatment, tapering, or discontinuation. Initial TCZ dosing included 8 mg/kg IV every 4 weeks,162 mg SC every 2 weeks, or 162 mg SC weekly	No untreated control	Over 12 months of TCZ treatment, FVC increased from 84.3±13.7% predicted to 88.5±16.4% predicted among 10 ILD patients with available data. After TCZ discontinuation, clinical worsening occurred in all 3 patients who stopped TCZ after a beneficial clinical course, including new ILD in one patient	FVC showed a statistically significant change at 12 months with p=0.04	TCZ was associated with FVC improvement and stabilization in the small cohort. However, this study primarily evaluated TCZ tapering and discontinuation rather than comparative ILD efficacy thus limiting the interpretation
Khanna et al., 2022 [28]	212 total; 136 with SSc-ILD at baseline: TCZ n=68 and placebo n=68 during the 48-week double-blind period. Open-label extension/week-96 comparison reported as continuous TCZ vs placebo-to-TCZ transition	Weekly 162mg of TCZ SC or weekly placebo injection	Placebo for 48 weeks, followed by open-label TCZ in the placebo-to-TCZ group; continuous TCZ group received TCZ through week 96	Baseline-to-week 96 FVC decline was smaller in the continuous TCZ group than in the placebo-to-TCZ group: -28.9 mL(-0.6% predicted) vs -157.6 mL(-4.1% predicted), respectively. DLCO % predicted changed by +1.3 in the continuous TCZ group vs -5.6 in the placebo-to-TCZ group	Statistical significance was not stated but the 95% CI is as follows: FVC for TCZ group: -3.1 to 2.0, FVC for placebo-to-TCZ group: -6.7 to -1.6, DLCO for TCZ group: -2.1 to 4.7, DLCO for placebo-to-TCZ group: -8.7 to -2.6	Continuous TCZ was associated with relative preservation of FVC compared with delayed TCZ initiation. Because week-96 data came from an open-label extension, findings should be interpreted as supportive of FVC stabilization rather than proof of causal improvement
Yilmaz et al., 2021 [29]	61 total; RTX n=27 and CY n=34	RTX courses every six months; each course included 2 doses of 500- 1000 mg IV two weeks apart	CY 1 g/month for six months, then spaced dosing based on response	At 24 months, change in FVC % predicted was +1.0±6.8 in CY group and +1.1±9.2 in RTX group. Change in DLCO % predicted was -1.7±16.9 in CY and +6.6±6.5 in RTX group	CY showed statistically significant within-group FVC % predicted improvement at selected earlier timepoints, but not at 24 months. RTX showed statistically significant within-group DLCO % predicted improvement at 24 months, p=0.019. These within-group findings do not establish significance between groups	CY showed selected earlier within-group FVC improvement, while RTX showed within-group DLCO improvement at 24 months. These findings suggest potential stabilization/improvement patterns but do not establish any therapeutic superiority
Roofeh et al., 2021 [12]	210 total; 136 with ILD: TCZ n=68, placebo n=68	TCZ 162 mg injection SC weekly for 48 weeks	Placebo for 48 weeks with background immunosuppressive therapy not permitted	At 48 weeks, the least squared means of FVC change was -0.1% for TCZ, and -6.3% for placebo. No changes were reported for DLCO	Between-group difference in least-squares mean FVC % predicted change favored TCZ over placebo, p<0.0001	TCZ was associated with preservation of FVC compared with placebo in early SSc-ILD. Findings support TCZ as a potential disease-stabilizing therapy, but should not be stated as PFT reversal
Robles-Perez et al., 2020 [30]	18 patients with CTD-ILD; 7 had SS-ILD	RTX 1 g IV on days zero and 14 every six months	No untreated control with ongoing treatment remaining unchanged	In the SSc-ILD subgroup, FVC and DLCO changes were numerically reported after RTX, but statistical testing was reported for the overall CTD-ILD cohort rather than the SSc-ILD subgroup specifically, limiting interpretation	SSc-ILD specific p-values NR. Overall CTD-ILD p-values should not be presented as SSc-ILD-specific significance. Overall CTD-ILD FVC p-values: at 1 year p=0.003; 2 years p=0.052. Overall CTD-ILD DLCO p-values: 1 year p<0.001; at 2 years p=0.001	RTX was associated with numeric pulmonary improvement in the small SSc-ILD subgroup, but SSc-ILD-specific statistical significance was NR. Conclusions should be limited because the study comprises mixed CTD-ILD outcomes
Sircar et al., 2018 [31]	64 total; but 60 analyzed due to four withdrawals: RTX n=30, CY n=30	RTX 1000 mg IV at days zero and 15	CY 500 mg/m^2^ IV every four weeks for 24 weeks	At six months, the FVC % predicted for the RTX and CY groups was 67.52±13.59 and 58.06±11.23, respectively. FVC volume was 1.65±0.47 L in RTX group vs 1.42±0.46 L in CY group	Within-group FVC improvement was significant in RTX: FVC % predicted p=0.002 and FVC volume p<0.001. CY changes were not significant: FVC % predicted p=0.496 and FVC volume p=0.356. Between-group estimates favored RTX for both FVC % predicted and FVC volume	RTX was associated with statistically significant within-group FVC improvement, and CY was not. Between-group estimates favored RTX, but the open-label design and modest sample size require interpretation carefully
Daoussis et al., 2017 [32]	51 total with SSc-ILD; RTX n=33 Control n=18	RTX 375 mg/m² IV weekly for four weeks. Cycles repeated every six months	Control group received standard immunosuppressive therapy, including azathioprine, MTX, MMF, and/or steroids	For the RTX group, FVC increased from 80.6±21.21% predicted at baseline to 86.90±20.56% predicted at two years and 91.60±14.81% predicted at seven years. DLCO was 59.22±18.17% predicted at baseline and 61.51±17.58% predicted at two years	RTX was associated with a mean FVC % predicted increase of 10.02 percentage points compared with control, p=0.004. DLCO increased by 8.14 percentage points compared with control, p=0.043. RTX within-group FVC p=0.041 at two years and DLCO p=0.053 at two years	RTX was associated with longer-term FVC stabilization and improvement compared with control. However, open-label design, concurrent therapies, and attrition bias at seven years limit certainty
Lepri et al., 2016 [33]	23 total with SSc-ILD	RTX; exact dose NR, but cumulative mean dose reported as 2.8 g at one year and 1.75 g at two years	No untreated control; concurrent DMARDs in some patients	FVC was 89% predicted (65-113; n=21) at one year and 74.5% predicted (50-91; n=10) at two years. DLCO was 61% predicted (47-64; n=19) at one year and 57.5% predicted (38.5-70.5; n=8) at two years	FVC change was not statistically significant at one year, p=0.98 or two years, p=0.066. DLCO change was not statistically significant at one year, p=0.62 or two years either, p=0.89	RTX was associated with pulmonary stabilization rather than clear improvement. FVC and DLCO changes were not statistically significant, and the interpretation is limited
Jordan et al., 2015 [34]	63 total SSc patients treated with RTX; ILD analysis compared 9 RTX-treated patients with 9 matched non-RTX controls.	RTX, most commonly two infusions of 1000 mg over two weeks	Matched non-RTX controls selected from EUSTAR database	At six months, FVC% predicted change was +0.8±2.2 in RTX vs -4.8±1.7 in matched controls. DLCO % predicted change was +3.7±1.4 in RTX vs +6.2±6.2 in matched controls	RTX within-group FVC change was not statistically significant, p=0.5. RTX within-group DLCO change was statistically significant, p=0.03	RTX may have helped stabilize FVC compared with matched controls in this very small, nested case-control analysis. The small subgroup and post hoc observational design limit the interpretation
Khanna et al., 2016 [35]	87 total SSc patients randomized to TCZ or placebo; ILD was not required for enrollment	TCZ 162 mg SC weekly	Placebo for 48 weeks, escape treatment with MTX, hydroxychloroquine, or MMF permitted after 24 weeks if deteriorating	FVC volume decline was smaller with TCZ group than placebo; -34 mL vs -171 mL at 24 weeks and -117 mL vs -237 mL at 48 weeks	Between-group FVC volume difference was 136 mL at 24 weeks (95% CI 9-264, p=0.0368) and 120 mL at 48 weeks (95% CI -23-262, p=0.0990)	TCZ was associated with significantly less FVC volume decline than placebo at 24 weeks. Because ILD was not required for enrollment, pulmonary findings should be interpreted carefully when applying them to clinically established SSc-ILD patients
Kuwana et al., 2024 [36]	20 patients randomized to TCZ or placebo followed by open-label TCZ from weeks 48-96.	TCZ 162 mg SC for 48 weeks followed by open-label TCZ	Placebo for 48 weeks followed by open-label TCZ.	Baseline-to-week-48 FVC % predicted change was +3.3 with TCZ vs -3.8 with placebo. DLCO % predicted change was +2.5 with TCZ vs -2.8 with placebo.	Exact p-values NR	TCZ appeared to preserve FVC and DLCO compared with placebo in the small SSc-ILD cohort, but the small sample and open label extension limit definitive conclusions

Forced Vital Capacity (FVC)

FVC was the most consistently reported pulmonary outcome across the studies. Overall, targeted immunomodulatory therapy was more frequently associated with improvement or stabilization in FVC. Within the studies evaluating TCZ, the placebo-controlled studies reported the strongest findings. In the focuSSced ILD subgroup, placebo-treated patients demonstrated FVC decline over 48 weeks, while patients given TCZ had a 7.06 percentage-point difference in predicted FVC change at the same interval [23]. Similarly, Roofeh et al. reported a least-squares mean FVC change of -0.1% in the TCZ group compared to -6.3% in the placebo group at 48 weeks [12]. In the open-label extension reported by Khanna et al., patients receiving subcutaneous TCZ had less overall FVC decline compared to the patients initially assigned to the placebo group [28]. A similar trend was reported by Kuwana et al., who noticed an FVC improvement in the TCZ group and an FVC decline in the placebo group at the end of the 48-week period [36]. Of note, some TCZ findings were derived from related trial cohorts, open-label extensions, or post-hoc subgroup analyses, which may amplify the apparent weight of evidence if interpreted as fully independent datasets; for this reason, this data must be interpreted cautiously. 

These trends did not hold true when discussing real-world comparative data. In the inverse probability of treatment weighting (IPTW)-adjusted analysis conducted by Yan et al., FVC change post-treatment did not significantly differ among TCZ, RTX, MMF, or CY groups one year after initiation [20]. Likewise, TCZ was not associated with a significant FVC change compared with standard of care controls at 12±3 months in a matched European Scleroderma Trials and Research (EUSTAR) cohort [26]. 

Studies evaluating RTX generally reported FVC stabilization, with some reporting statistically significant improvements. Hiura et al. reported no significant difference in FVC change between RTX and CY at six or 12 months [17]. Conversely, in the sytemic sclerosis subgroup within the RECITAL trial, RTX and CT were associated with similar FVC improvement values at 24 and 48 weeks [11]. Sircar et al. reported a significant within-group improvement in FVC for the RTX group but an insignificant within-group change in the CY group at six months [31]. Of note, this should not be interpreted as definitive superiority unless a between-group analysis is completed. Yilmaz et al. evaluated RTX and CY over 24 months, reporting a significant within-group FVC improvement in the CY group only [29]. Kuzumi et al. reported significant improvement in predicted FVC after repeated RTX doses, although the study served more so as a long-term extension of the DESIRES trial [10]. Moazedi-Fuerst et al. reported stable predicted FVC during the first year of RTX therapy, with no report of FVC decline [21]. Other cohorts, too, reported stable or numerically improved FVC values following RTX treatment, though insignificant [25,30,32-34]. 

Combination therapy was sparsely analyzed across the included studies. In a retrospective cohort study by Barde et al., the combination of MMF/RTX was associated with a significant FVC improvement at 12 months, while RTX and MMF monotherapy were not [18]. This finding suggests a possibility for additive benefit, although the retrospective observational design limits interpretation. JAKi evidence was also limited. Junfei et al. evaluated tofacitinib therapy, noting that it did not significantly improve FVC compared with conventional immunosuppressives (either CY, MMF, azathioprine, methotrexate, or tripterygium glucosides) [24]. Based on the available data, JAKi therapy cannot be interpreted as having an established pulmonary function benefit in SSc-ILD patients. 

DLCO and TLC 

DLCO and TLC were less consistently reported than FVC in the included studies. Similar to FVC, DLCO generally showed stabilization after exposure to immunomodulatory therapy, although statistically significant improvement was less consistently observed. When DLCO was evaluated following both MMF/RTX combination and monotherapy, there was numerical improvement, although not statistically significant [18]. This was held true in the study by Yan et al., where DLCO changes were not statistically significant when compared between groups receiving MMF, RTX, TCZ, and CY [20]. Eight of the studies evaluating RTX did not compare it to other immunomodulating therapies, out of which two demonstrated statistical significance. In the study by Robles-Perez et al., FVC and DLCO numerically improved after RTX, however the p-values corresponded to the overall CTD-ILD cohort rather than the SSc-ILD group specifically [30]. Goldman et al. reported stabilization of DLCO trajectories following RTX and TCZ initiation [19]. In the RECITAL trial, which compared RTX and CY, there were similar changes to DLCO between groups at 24 and 48 weeks [11]. Yilmaz et al. also compared CY and RTX, with a statistically significant improvement in DLCO within two years of RTX treatment [29]. Jordan et al. compared the RTX treatment group to matched controls in the EUSTAR database with a statistically significant change in DLCO [34]. 

Junfei et al. assessed tofacitinib, reporting a six-month DLCO improvement; this was one of the few studies to report a TLC change at six months, although insignificant [24]. In the focuSSed trial, patients who received TCZ throughout demonstrated a greater improvement in DLCO compared to the placebo-TCZ group [23]. Similarly, Kuwana et al. found that the DLCO of the patients receiving continuous TCZ treatment demonstrated greater lung function preservation compared to the placebo-TCZ group [36]. 

Overall, DLCO demonstrated stabilization as opposed to obvious improvement with immunomodulatory therapies, with RTX therapy most consistently being significant. TCZ demonstrated stabilized DLCO over time suggesting avenues for future treatment and room for future research. TLC was rarely reported and was insignificant when it was. Although there were findings demonstrating stabilization of DLCO, interpretation was limited due to variable outcome reporting and potential confounding from comorbid cardiopulmonary disease. There is no reasonable conclusion that can be drawn for TLC due to the lack of reporting. 

HRCT and Radiographic Progression 

Radiographic outcomes were less consistently reported compared to pulmonary function outcomes. When radiographic outcomes were reported, findings generally supported disease stabilization rather than improvement. Roofeh et al. reported that baseline levels of quantitative ILD (qILD) defined as “ground glass opacities, honeycombing, and fibrotic reticulation” are inversely proportional to FVC values; there was a significant improvement of qILD by week 48 of TCZ treatment, especially in patients with a higher baseline qILD score [12]. Hiura et al. reported progression of pulmonary fibrosis in three out of 20 patients treated with RTX and five out of 30 patients treated with CY, but these findings were insignificant [17]. In contrast, the study by Junfei et al. demonstrated significant improvement in HRCT scores for the tofacitinib cohort despite nonsignificant improvement in PFTs [24]. Some studies were limited by missing or inconsistently reported data, such as in the MMF/RTX combination cohort evaluated by Barde et al. [18]. Others provided baseline HRCT findings to support ILD diagnosis, but did not report serial HRCT outcomes post-exposure [19,21,22]. While the radiographic data provided possibly suggests disease stabilization, the evidence remains less robust secondary to inconsistent reporting. 

Overall Pulmonary Response 

The included studies suggested that targeted immunomodulation, particularly with RTX or TCZ, is associated with pulmonary function stabilization in SSc-ILD. TCZ had the strongest placebo-controlled evidence for FVC preservation, while RTX demonstrated consistent stabilization across comparative studies and retrospective cohorts, when compared to CY. Combination MMF/RTX therapy also showed potential benefit in one cohort, whereas evidence for JAKi remained limited and primarly radiographic rather than validated by PFTs. Comparative efficacy, however, remains uncertain. Many of the included studies were retrospective, sub-group based, or post-hoc. In addition, concomitant baseline immunosuppressive therapy varied significantly, as did follow up duration, outcome reporting, and HRCT evaluation. Ultimately, these factors limited direct comparison across studies and precluded quantitative pooling. Out of the available evidence, there is support for targeted immunomodulation as a potential disease-stabilizing approach in SSc-ILD, but the findings do not establish superiority of one agent over another. 

Discussion 

Across the reviewed literature, the findings did not necessarily support a dramatic reversal or improvement of lung disease, but rather an attenuation of progression intensity and speed, particularly when it came to FVC. This is a clinically important distinction to consider. That is, in a progressive fibrosing disease like SSc-ILD, with the propensity to become irreversible, stabilization of pulmonary function may be significantly desired by clinicians and patients [39]. The findings suggest that SSc-ILD management might be better approached through the lens of disease stabilization rather than overt pulmonary improvement or disease reversal. 

Historically, therapies like CY and MMF have been used to slow disease progression; notable trials, including Scleroderma Lung Study I and II, established that immunosuppression can modify the course of SSc-ILD, while also emphasizing the moderate magnitude of benefit, toxicity concerns, and limitations associated with conventional therapy [5]. More recently, SENSCIS reframed therapeutic success in SSc-ILD, supporting the broader concept that stabilization is a meaningful endpoint in progressive fibrosing lung disease [40]. The apparent preservation of FVC seen with TCZ and overall stabilization seen with RTX may be clinically relevant in future conversations regarding management options for SSc-ILD patients. TCZ demonstrated the clearest FVC preservation outcomes when discussing placebo-controlled studies. This may be because of the IL-6 signaling in the pathogenesis of systemic sclerosis, indicating that immune activation and fibrotic signaling may still be modifiable early in the disease course; this apparent benefit of TCZ raises the possibility that timing and phenotype are important determinants of treatment response [12,41]. However, the less definitive findings from real-world comparative populations demonstrate the challenge of translating study results into broader clinical practice. Observational populations often include patients with more variable disease duration, variable background immunosuppression, and comorbidities, all of which may dilute or obscure treatment effects.

RTX also demonstrated consistent pulmonary stabilization; however, not all of the studies included a control group, thereby decreasing the certainty of therapeutic efficacy. Given the role of B cells in autoantibody production, antigen presentation, and fibroblast activation, the clinical rationale for physicians to administer RTX for SSc-ILD is quite compelling [42]. However, the data must be interpreted carefully as many of the RTX cohorts continued background chronic treatment with concomitant MMF, steroids, or other immunosuppressive therapies, allowing for potential confounding. Because of this, and without an untreated control group, it is difficult to say whether pulmonary function preservation was attributable to RTX independent of concurrent background therapy. When it came to JAKi therapy, the findings remained inconclusive. The discordance between radiographic improvement and significant PFTs within the selected literature may suggest that radiographic alterations can precede physiologic improvement.

Future studies investigating JAKi may benefit from including standardized HRCT scoring and having a longer follow-up duration to determine whether early changes seen on HRCT translate into clinically meaningful PFT alterations. Roofeh et al. reported that TCZ stabilized FVC regardless of baseline qILD severity, leading the authors to suggest that patients should receive treatment earlier in the disease course to prevent future complications [12]. FVC remained the most interpretable and consistently reported outcome, whereas DLCO, TLC, and HRCT outcomes were more difficult to interpret. FVC reporting is crucial to SSc-ILD trials because it is reproducible, clinically meaningful, and associated with disease progression [43]. While DLCO is still clinically important, it is less specific in SSc-ILD because it can also be influenced by comorbid pulmonary vascular disease, anemia, and cardiovascular disease. Thus, DLCO fluctuations should not be interpreted in isolation [44]. 

There were various limitations within the studies assessed. The majority of studies were retrospective or observational, increasing the risk of confounding and selection bias. Some studies lacked an untreated control or a placebo group, which made it difficult to determine whether the outcome of the immunomodulator was improvement or stabilization. Background immunosuppressive therapy varied, limiting attribution of treatment effect to the targeted immunomodulator itself. A prevalent limitation exhibited by multiple studies was a small sample size for SSc-ILD patients. For example, in Robles-Perez et al., the sample size was 18 total patients out of whom only seven had systemic sclerosis [30]. FVC was reported across most studies, but other metrics such as DLCO, TLC, and HRCT findings were inconsistently reported. Additionally, several studies enrolled broader systemic sclerosis populations or did not require overt ILD evidence at baseline, meaning that pulmonary outcomes from these trials may not fully reflect treatment response in patients with clinically established SSc-ILD. The PFT dispersion metrics varied between studies, as did the availability of p-values, confidence intervals, effect estimates to signify statistical significance. While stability of PFT parameters is suggested by the study's outcomes, larger randomized control trials are needed to validate the strength of the conclusions. 

A strength of this paper is its realistic focus on stabilization of PFT parameters instead of emphasizing overt statistically significant improvement. This mirrors real-world outcomes in which patients may be trying to prevent worsening of their lung function, rather than looking for overt improvement. There is also an array of immunomodulatory therapies covered, demonstrating the evolution of treatment options available for SSc-ILD. There are also clear distinctions between placebo-controlled results versus real-world observational studies, demonstrating that not all studies should be interpreted in the same way. For this reason, a narrative synthesis was appropriate since the included studies varied widely in intervention and outcome reporting. By identifying gaps in existing literature, this paper emphasizes areas for future research. 

Future directions for research should prioritize the gaps identified, namely having a large SSc-ILD sample size and completing prospective SSc-ILD-specific comparative trials. Patients should be categorized according to baseline characteristics such as disease duration, baseline PFTs, antibody status, and concurrent immunosuppressive therapies to tailor specific therapies. Future research should also standardize HRCT scoring and utilize predefined radiographic progression criteria to assess outcomes. Studies should also evaluate immunomodulatory monotherapy versus combination therapy with antifibrotic agents and anti-inflammatory agents to evaluate the effectiveness of combined treatment modalities that address both the inflammatory and fibrotic mechanisms seen in SSc-ILD. 

## Conclusions

In this systematic review, target immunomodulation was generally associated with stabilization of pulmonary function in SSc-ILD, specifically with respect to FVC changes observed before and after therapy initiation. TCZ demonstrated the most consistent placebo-controlled evidence for FVC preservation, while RTX recurrently showed disease stabilization across comparative and observational studies. Evidence of JAKi efficacy remained preliminary, with notable radiographic improvement reported among selected studies, although data were quite limited, and no significant change was seen in PFT outcomes. These findings must be interpreted cautiously. There was substantial heterogeneity amongst the selected studies, with variation in study design, concomitant immunosuppressive therapy, comparator groups, and outcome reporting. Many of the studies were retrospective, limiting the ability to infer causality. The current evidence supports targeted immunomodulation as a potential disease-stabilizing approach to SSc-ILD management but does not clarify a superior agent or standardized treatment criteria. In the future, prospective SSc-ILD trials are necessary to clarify specific therapies, temporal treatment guidelines, and to evaluate potential combination strategies. 

## Appendices

Appendix A 

**Table 5:** Search strategy for EMBASE

Search	Keywords/Search Terms	Results
1	systemic sclerosis' OR 'scleroderma'	69,901
2	interstitial lung disease' OR ild OR 'pulmonary fibrosis' OR 'lung fibrosis'	127,469
3	1 AND 2	12,774
4	janus kinase inhibitor*' OR 'jak inhibitor*' OR 'interleukin-6 inhibitor*' OR 'il-6 inhibitor*' OR 'rituximab' OR 'b cell depletion' OR 'anti-cd20' OR 'tofacitinib' OR 'baricitinib' OR 'upadacitinib' OR 'filgotinib' OR 'tocilizumab' OR 'sarilumab' OR 'ocrelizumab' OR 'ofatumumab'	217,331
5	forced vital capacity' OR fvc OR dlco OR 'diffusing capacity of the lung for carbon monoxide' OR hrct OR 'high-resolution computed tomography' OR 'pulmonary function test*' OR pft* OR 'lung function'	267,159
6	3 AND 4	1,487
7	5 AND 6	768

Appendix B 

**Table 6:** Search strategy for OVID Medline

Search	Keywords/Search Terms	Results
1	exp Systemic Sclerosis/ OR (systemic sclerosis OR scleroderma).mp.	38,094
2	exp Interstitial Lung Diseases/ OR (interstitial lung disease OR ILD OR pulmonary fibrosis OR lung fibrosis).mp.	113,469
3	1 AND 2	5,070
4	exp Rituximab/ OR exp Tocilizumab/ OR exp Sarilumab/ OR exp Janus Kinase Inhibitors/ OR (janus kinase inhibitor* OR JAK inhibitor* OR interleukin-6 inhibitor* OR IL-6 inhibitor* OR rituximab OR b cell depletion OR anti-cd20 OR tofacitinib OR baricitinib OR upadacitinib OR filgotinib OR tocilizumab OR sarilumab OR ocrelizumab OR ofatumumab).mp.	61,458
5	3 AND 4	273
6	exp Lung Function Tests/ OR exp Tomography, X-Ray Computed/ OR (forced vital capacity OR FVC OR DLCO OR diffusing capacity of the lung for carbon monoxide OR HRCT OR high-resolution computed tomography OR pulmonary function test* OR PFT* OR lung function).mp.	837,482

Appendix C 

**Table 7:** Search strategy for PubMed

Search	Keywords/Search Terms	Results
1	"Systemic Sclerosis"[Mesh] OR "systemic sclerosis" OR scleroderma	38,477
2	"Interstitial Lung Diseases"[Mesh] OR "interstitial lung disease" OR ILD OR "pulmonary fibrosis" OR "lung fibrosis"	61,452
3	1 AND 2	4,691
4	("Rituximab"[Mesh] OR "Tocilizumab"[Mesh] OR "Janus Kinase Inhibitors"[Mesh] OR "CD20 Antigens"[Mesh] OR rituximab OR ocrelizumab OR ofatumumab OR "b cell depletion" OR "B-cell depletion" OR anti-cd20 OR "anti-CD20" OR tocilizumab OR sarilumab OR tofacitinib OR baricitinib OR upadacitinib OR filgotinib OR "JAK inhibitor*" OR "interleukin-6 inhibitor*" OR "IL-6 inhibitor*")	62,524
5	3 AND 4	240

Appendix D 

**Table 8:** Search strategy for Web of Science

Search	Keywords/Search Terms	Results
1	("systemic sclerosis" OR scleroderma) AND ("interstitial lung disease" OR ILD OR "pulmonary fibrosis" OR "lung fibrosis") AND (rituximab OR ocrelizumab OR ofatumumab OR "b cell depletion" OR "B-cell depletion" OR anti-cd20 OR "anti-CD20" OR tocilizumab OR sarilumab OR tofacitinib OR baricitinib OR upadacitinib OR filgotinib OR "JAK inhibitor*" OR "interleukin-6 inhibitor*" OR "IL-6 inhibitor*"))	435
